# A High-Fat Diet Increases the Characteristics of Gut Microbial Composition and the Intestinal Damage Associated with Non-Alcoholic Fatty Liver Disease

**DOI:** 10.3390/ijms242316733

**Published:** 2023-11-24

**Authors:** Xiaoyang Zhu, Jiajia Cai, Yifu Wang, Xinyu Liu, Xiaolei Chen, Haifei Wang, Zhengchang Wu, Wenbin Bao, Hairui Fan, Shenglong Wu

**Affiliations:** 1 Key Laboratory for Animal Genetics, Breeding, Reproduction and Molecular Design, College of Animal Science and Technology, Yangzhou University, Yangzhou 225009, China; mz120211472@stu.yzu.edu.cn (X.Z.); mz120211445@stu.yzu.edu.cn (Y.W.); mz120231591@stu.yzu.edu.cn (X.L.); mx120230917@stu.yzu.edu.cn (X.C.); hyfiwang@yzu.edu.cn (H.W.); zcwu@yzu.edu.cn (Z.W.); wbbao@yzu.edu.cn (W.B.); 2 Joint International Research Laboratory of Agriculture and Agri-Product Safety, The Ministry of Education of China, Yangzhou University, Yangzhou 225009, China; jjcai@yzu.edu.cn; 3 Institute of Comparative Medicine, College of Veterinary Medicine, Yangzhou University, Yangzhou 225009, China

**Keywords:** non-alcoholic fatty liver disease, 16S rRNA-seq, gut microbiota, biodiversity, gut barrier

## Abstract

The prevalence of non-alcoholic fatty liver disease (NAFLD) is increasing annually, and emerging evidence suggests that the gut microbiota plays a causative role in the development of NAFLD. However, the role of gut microbiota in the development of NAFLD remains unclear and warrants further investigation. Thus, C57BL/6J mice were fed a high-fat diet (HFD), and we found that the HFD significantly induced obesity and increased the accumulation of intrahepatic lipids, along with alterations in serum biochemical parameters. Moreover, it was observed that the HFD also impaired gut barrier integrity. It was revealed via 16S rRNA gene sequencing that the HFD increased gut microbial diversity, which enriched *Colidextribacter*, *Lachnospiraceae-NK4A136-group*, *Acetatifactor*, and *Erysipelatoclostridium*. Meanwhile, it reduced the abundance of *Faecalibaculum*, *Muribaculaceae*, and *Coriobacteriaceae-UCG-002*. The predicted metabolic pathways suggest that HFD enhances the chemotaxis and functional activity of gut microbiota pathways associated with flagellar assembly, while also increasing the risk of intestinal pathogen colonization and inflammation. And the phosphotransferase system, streptomycin biosynthesis, and starch/sucrose metabolism exhibited decreases. These findings reveal the composition and predictive functions of the intestinal microbiome in NAFLD, further corroborating the association between gut microbiota and NAFLD while providing novel insights into its potential application in gut microbiome research for NAFLD patients.

## 1. Introduction

Non-alcoholic fatty liver disease (NAFLD) has emerged as a paramount global health concern, representing a significant covert condition that escalates the susceptibility to various chronic ailments, including type 2 diabetes and cardiovascular diseases [1]. The progression of NAFLD in patients may potentially result in the development of steatohepatitis, liver cirrhosis, and even hepatocellular carcinoma [2]. This rising disease prevalence will create a growing economic and public health burden [3]. At present, no pharmacological interventions have been approved for the management of NAFLD, and the prevailing clinical recommendation is to initiate lifestyle modifications, including weight reduction through dietary adjustments and consistent engagement in physical activity [4]. The Mediterranean diet is recommended as one of the regimens for the prevention and treatment of NAFLD [5], given its demonstrated efficacy in reducing hepatic steatosis and improving insulin sensitivity [6]. Additionally, other dietary approaches such as ketogenic, high-protein, plant-based, low-carbohydrate, and intermittent fasting strategies have also exhibited promising outcomes in terms of liver health [7].

The latest research findings have revealed notable dissimilarities in the composition of gut microbiota in individuals with fatty liver. A study on pediatric subjects revealed that NAFLD was associated with higher levels of the phyla Bacteroidetes and Proteobacteria, while children who were simply overweight or obese had elevated levels of the phylum Firmicutes [8]. Meanwhile, the transplantation of gut microbiota isolated from donors with diet-induced obesity to germ-free animals resulted in a significant augmentation of body weight and metabolic syndrome in recipient mice [9]. The administration of probiotics like *Lactobacillus lactis* and *Pediococcus* to HFD-induced NAFLD mice for 8 weeks improved NAFLD symptoms, suggesting a critical role of intestinal microbiota in NAFLD [10]. Increasing evidence has proved that NAFLD occurrence and development are driven by gut–liver axis unbalance and metabolites of gut microbiota [11]. Obese mice were observed to exhibit increased intestinal bacterial fermentation capacity and higher levels of short-chain fatty acids (SCFAs) compared to normal mice [12,13,14]. Gut microbiota derived factors and SCFAs (e.g., acetate, propionate, and butyrate) can have anti-inflammatory properties, which could prevent the progression of NAFLD [12]. A disruption in liver energy and substrate metabolism may arise from abnormalities in the composition and/or functioning of gut microbiota [15]. For instance, trimethylamine is a byproduct of choline metabolism by the gut microbiota; it can be oxidized into trimethylamine N-oxide, which has been implicated in exacerbating the development of NAFLD [16]. The gut microbiota, which produces proinflammatory lipopolysaccharide (LPS) and further influences hepatic lipid metabolism, has emerged as a crucial player in the development of NAFLD [17]. Additionally, bile acid metabolism by gut microbiota activates the nuclear bile acid receptor farnesoid X receptor signaling, thereby affecting the progression of NAFLD [18]. Therefore, the gut microbiota affects liver lipid metabolism through multiple mechanisms, participating in and promoting the formation of NAFLD. However, the function of the gut microbiota in development of NAFLD remains unclear and requires further investigation.

In the present study, 16S rRNA gene amplicon sequencing was applied to investigate the relationship between changes in gut microbiota structure and NAFLD. Thus, it provides new experimental data for the study of NAFLD diagnosis of biomarkers and treatment.

## 2. Results

### 2.1. High-Fat Diet (HFD) Triggered Weight Gain and Lipid Accumulation

**Figure 1:**
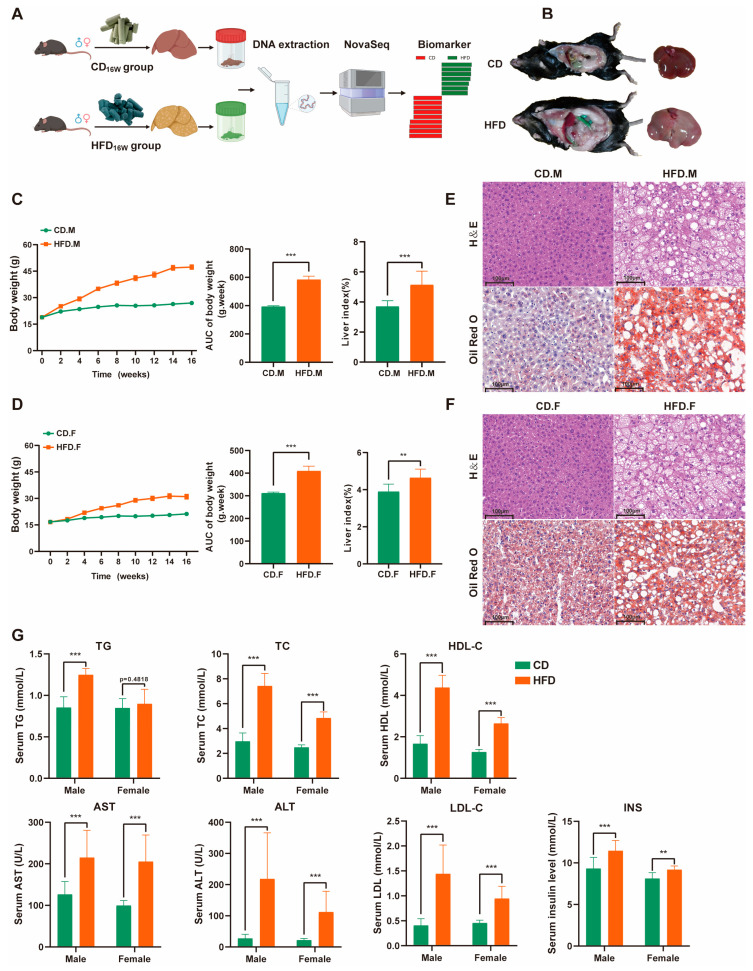
A comparative investigation into the physiological and pathological effects of a high-fat diet on male and female mice. (**A**) Experimental and data analysis workflow: an overview. (**B**) Representative pictures of CD and HFD mice, showing enormous discrepancy in body and liver size. (**C**,**D**) Body weight and liver index changes in CD or HFD fed mice over 16 weeks. (**E**,**F**) H&E and Oil red O of hepatic tissues from CD or HFD mice. Scale bar: 100 µm. (**G**) Levels of blood biochemical markers (TG, TC, HDL-C, AST, ALT, LDL-C, INS). Asterisks are employed to denote statistically significant differences (unpaired two-tailed Student’s *t*-test, ** indicates *p*-value < 0.01, *** indicates *p*-value < 0.001, ns indicates *p*-value > 0.05 *n* = 6–8 mice per group). HFD: high-fat diet; CD: chow diet.

By the conclusion of the 16-week HFD feeding period, a significant increase in average body weight was observed among male and female mice in the HFD group, as compared to the control group (Figure 1B). Additionally, there was a notable elevation in liver weight and liver index within the HFD group (Figure 1C,D). The histology of HFD mice livers, as demonstrated through H&E and Oil Red O staining, revealed significant macrovesicular steatosis in hepatocytes, slight ballooning degeneration, and lobular inflammation (Figure 1E,F). The biochemical analysis revealed significantly elevated levels of serum triglyceride (TG), total cholesterol (TC), alanine aminotransferase (ALT), aspartate aminotransferase (AST), high-density lipoprotein (HDL-C), low-density lipoprotein (LDL-C), and insulin (INS) in mice with NAFLD compared to the control group, as shown in Figure 1G. These findings indicate the successful establishment of an NAFLD mouse model.

### 2.2. HFD Enhanced Biodiversity and Altered Gut Microbial Composition

**Figure 2:**
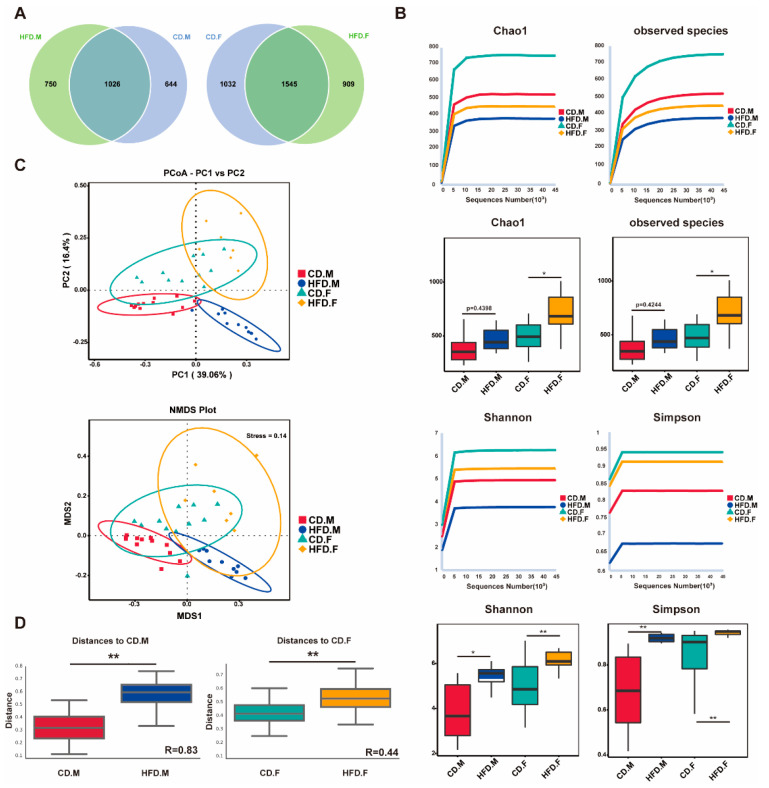
The gut microbiota in the cecal contents of CD and HFD mice was subjected to alpha and beta diversity analysis. (**A**) Composition of the gut microbiota of CD and HFD mice. (**B**) The alpha diversity was assessed using Chao1, Observed species, Shannon, and Simpson indices in both CD and HFD mice. (**C**) The beta diversity was evaluated through employing PCoA and NMDS based on Bray-Curtis distance matrices between CD and HFD mice. (**D**) Significance of community structure differences between groups were tested via Anosim analysis. * indicates *p*-value < 0.05, ** indicates *p*-value < 0.01 (Wilcox Rank-Sum test).

In this study, we characterized the gut microbiota composition through sequencing the v3–v4 region of the 16S rRNA gene. The Venn diagram is employed to illustrate the distribution and disparities of operational taxonomic units (OTUs) between HFD mice and CD mice (Figure 2B). The Chao1 and Observed-species indices were used to evaluate microflora richness, while the Shannon and Simpson indices provided comprehensive measures of both richness and evenness. The alpha diversity indices, including Chao1, Observed-species, Shannon, and Simpson, exhibited significant differences between the CD or HFD groups in the present study. However, Chao1 and Observed-species did not differ significantly in the male group. These results indicate that HFD can increase intestinal microflora richness/diversity, with female mice having greater species diversity than male mice.

Beta diversity analysis is used to examine changes in the overall structure of the gut microbiota. Principal Coordinate Analysis (PCoA) based on Bray–Curtis distance allows for the observation of dissimilarities between individuals or populations. In addition, non-metric multidimensional scaling (NMDS) was used to reveal non-linear structural relationships within ecological data across samples through representing bacterial community structure as points in a multidimensional space. PCoA and NMDS revealed that the distribution regions for gut samples from CD.M and HFD.M were separated in the two-dimensional coordinate system; however, the distribution regions for gut samples from CD.F and HFD.F partially overlapped (Figure 2C). Anosim analysis was performed to test whether group differences were significantly greater than within-group differences, with results indicating significant differences between groups (R > 0, *p* < 0.001) (Figure 2D). These results indicated that under normal conditions, female mice had an increased gut microbiota richness compared to male mice. Under HFD, male HFD mice showed an increase in gut microbiota richness, whereas female HFD mice showed a decrease in gut microbiota richness.

### 2.3. Relative Abundance of Bacteria at Phylum Level in the Intestinal Tract of CD and HFD Mice

**Figure 3:**
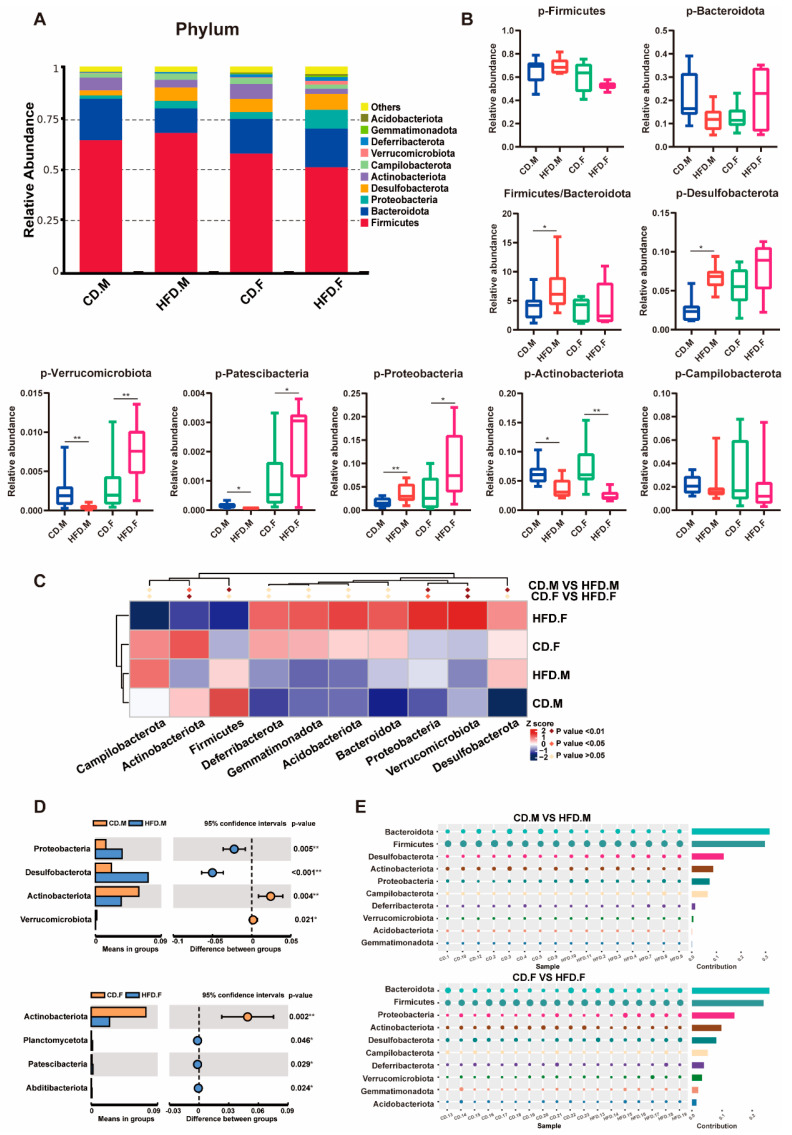
Phylum-level relative abundance of gut microbiota. (**A**) The stacked column graph displays the top 10 phyla with the highest relative abundance. (**B**) The effects of an HFD on the relative abundances of several intestinal flora were analyzed at the phylum level using box plots. (**C**–**E**) Through employing *t*-tests, Metastable complex heat maps, and Simper tests, we conducted an analysis at the phylum level to investigate the impact of an HFD on the composition of the gut microbiota. * indicates *p*-value < 0.05, ** indicates *p*-value < 0.01 (*t*-test).

Different diets shape the composition of the gut microbiota. We screened the 10 most abundant phyla and presented the results in a stacked column plot (Figure 3A). Among them, *Firmicutes* and *Bacteroidetes* are dominant taxa. Compared to the CD group of mice, the HFD-fed mice showed a higher *Firmicutes/Bacteroidetes* ratio (F/B). The statistical results for the gut microbiota with altered relative abundances were visualized using boxplots, and a *t*-test was conducted (Figure 3B). Metastat analysis revealed a significant decrease in the relative abundance of *Actinobacteriota* and *Verrucomicrobia* in male mice fed an HFD, accompanied by an increase in the relative abundance of *Desulfobacterota* and *Proteobacteria* when compared to the control group. In the HFD-fed female mice group, a significant decrease was observed in the relative abundance of *Actinobacteria*, whereas a significant increase was noted in the relative abundance of *Proteobacteria* and *Verrucomicrobia* (Figure 3C). Based on *t*-test analysis, four phyla were found to be significantly different in the male group: Proteobacteria, *Desulfobacterota*, *Actinobacteriota*, *and Verrucomicrobia*. In the female group, four phyla were found to be significantly different: Actinobacteriota, *Planctomycetota*, *Patescibacteria*, and *Abditibacteriota* (Figure 3D). The Simper analysis provides a quantitative assessment of the contribution of each species to the dissimilarities between the two groups (Figure 3E). The findings suggest that *Firmicutes*, *Bacteroides*, *Proteobacteria*, *Desulfobacterota*, Actinobacteriota, and *Verrucomicrobia* are likely to play a significant role in driving differentiation in male mice. In contrast, female mice indicate that the primary drivers of differentiation may include *Firmicutes*, *Bacterbia*, *Actinobacteriota*, *Verrucomicrobia*, *Proteobacteria*, *Planctomycetota*, *Patescibacteria*, and *Abditibacteriota.*

### 2.4. Relative Abundance of Bacteria at Genus Level in the Intestinal Tract of CD and HFD Mice

**Figure 4:**
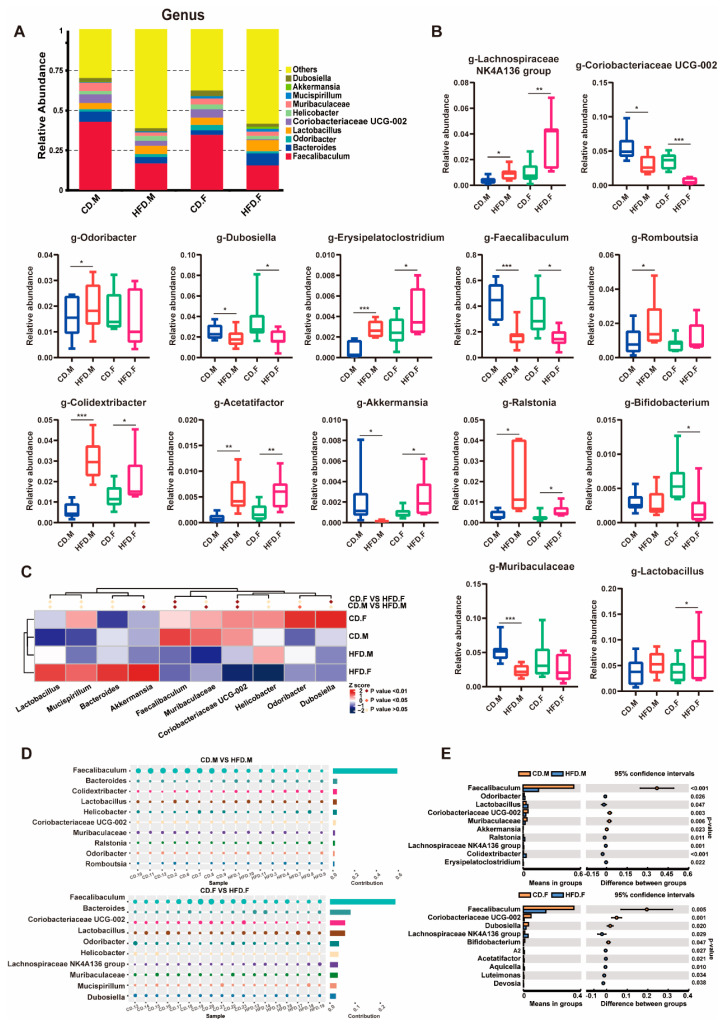
Genus-level relative abundance of gut microbiota. (**A**) The stacked column graph displays the top 10 genera with the highest relative abundance. (**B**) The effects of an HFD on the relative abundances of several intestinal flora were analyzed at the genus level using box plots. (**C**–**E**) Through employing *t*-tests, Metastable complex heat maps, and Simper tests, we conducted an analysis at the genus level to investigate the impact of an HFD on the composition of the gut microbiota. * indicates *p*-value < 0.05, ** indicates *p*-value < 0.01, *** indicates *p*-value < 0.001 (*t*-test).

At the genus level, discernible variations in gut microbiota composition and abundance exist between CD and HFD mice. A stacked bar chart analysis was conducted on the top 10 genera with the highest abundance (Figure 4A). Box plots were used to display statistical results for gut microbiota whose relative abundance changed during *t*-test (Figure 4B). Metastat analysis showed that male and female mice fed an HFD had significantly reduced relative abundances of *Faecalibaculum*, *Coriobacteriaceae-UCG-002*, *Muribaculaceae*, *Akkermansia*, and *Dubosiella*, while the relative abundance of *Odoribacter* was increased. However, in female mice fed an HFD, the relative abundance of *Akkermansia* was significantly increased (Figure 4C). *T*-test analysis showed that the main differential genera of males included *Faecalibaculum*, *Odoribacter*, *Lactobacillus*, *Coriobacteriaceae-UCG-002*, and *Muribaculacea*. In the female group, the differential genera were mainly *Faecalibaculum*, *Coriobacteriaceae-UCG-002*, *Dubosiella*, *Lachnospiraceae-NK4A136 group*, and *Bifidobacterium* (Figure 4E) (*p* < 0.05). The Simper analysis showed that the top five contributing genera in the male mice group were *Faecalibaculum*, *Bacteroides*, *Colidextribacter*, *Lactobacillus,* and *Helicobacter*. In the female mice group, the top five contributing genera were Faecalibaculum, *Bacteroides*, *Coriobacteriaceae-UCG-002*, *Lactobacillus*, and *Odoribacter* (Figure 4D). The gut microbiota composition at the genus level in mice was significantly altered following a 16-week HFD intervention, while gender differences also exerted a notable impact on the gut microbiota structure of HFD-fed mice.

### 2.5. Biomarkers of Gut Microbiota in the Intestinal Tract of Mice with NAFLD

**Figure 5:**
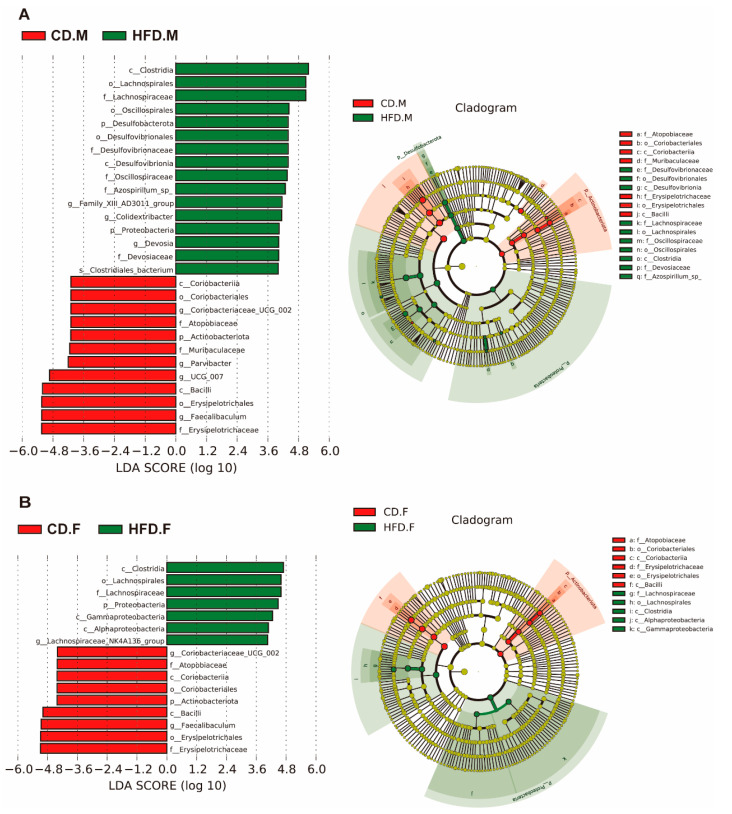
The identification of functional biomarkers using linear discriminant analysis effect size (LEfSe). (**A**) The intestinal microbiota in male mice subjects was subjected to LEfSe analysis. (**B**) The intestinal microbiota in female mice subjects was subjected to LEfSe analysis.

The relative abundance of different gut microbiota taxa (from phylum to genus) in the samples was evaluated using linear discriminant analysis effect size (LEfSe). As shown (Figure 5A,B), the red bars represent bacterial groups enriched in the CD group, while the green bars represent bacterial groups enriched in the HFD group. Species with LDA scores >4 are considered biomarkers. The results show that in the male group fed with an HFD, a total of 28 biomarkers were identified, with 16 significantly enriched in the HFD group and 12 significantly enriched in the CD group. In the female group, 16 biomarkers were identified, with 7 significantly enriched in the HFD group and 8 significantly enriched in the CD group. After the HFD, biomarkers shared by both male and female groups showed significant changes. Specifically, the levels of *Clostridia*, *Lachnospirales*, *Lachnospiraceae*, and *Proteobacteria* were significantly increased. Conversely, *Eyrsipelotrichaceae*, *Erysipelotrichales*, *Faecalibaculum*, and *Bacilli* exhibited a significant decrease. These results indicate that these alterations in gut microbiota are likely to be associated with the development of NAFLD and may be potential novel biomarkers for NAFLD.

### 2.6. Correlation Analysis between Gut Microbiota and Indicators Related to NAFLD

**Figure 6:**
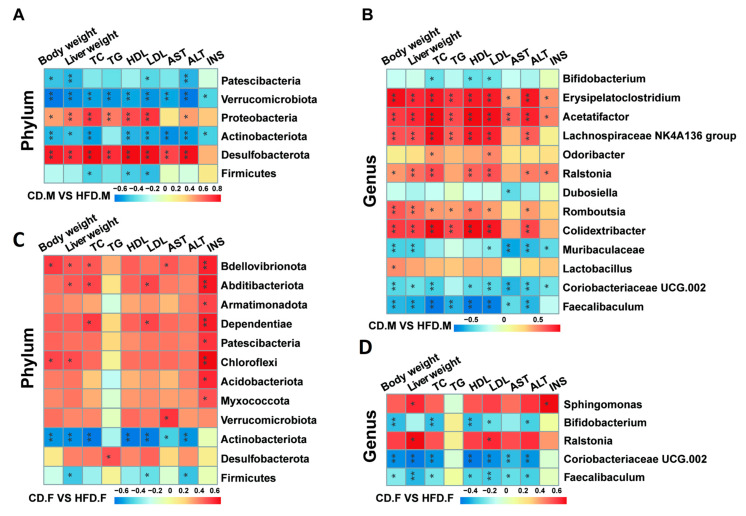
Integrated analysis of gut microbiota composition and NAFLD-associated markers. (**A**) Differential bacteria at phylum level in male group. (**B**) Differential bacteria at genus level in male group. (**C**) Differential bacteria at phylum level in female group. (**D**) Differential bacteria at genus level in female group. Spearman rank correlations were determined between the altered gut microbiota and serum levels of TG, TC, AST, ALT, INS, LDL-C, and HDL-C. The color red indicates a positive correlation while blue represents a negative correlation. Significant correlations are indicated by * *p*-value < 0.05, ** *p*-value < 0.01 marked with asterisks.

To further elucidate the mutual relationship between gut microbiota and the progression of NAFLD, Spearman’s rank correlation analysis was employed to investigate the association between the top 20 gut microbiota and parameters such as body weight, liver weight, and serum markers related to NAFLD in mice. The results were presented in the form of a heatmap, revealing several microorganisms closely associated with NAFLD features. After 16 weeks of HFD induction, the results indicate that, compared to female mice, male mice showed stronger correlations between body weight, liver weight, NAFLD-related serum markers, *Desulfobacterota*, *Verrucomicrobiota*, *Proteobacteria*, and *Patescibacteria* at the phylum level (Figure 6A,C). This study also found negative correlations between *Verrucomicrobiota*, *Patescibacteria*, and NAFLD-related markers in male mice, while these correlations were positive in female mice. Additionally, this study observed a stronger positive correlation between INS and most gut microbiota in female mice, whereas in male mice, INS showed a significant negative correlation with *Actinobacteriota* and *Verrucomicrobiota*. At the genus level, male mice showed stronger correlations between body weight, liver weight, NAFLD-related serum markers, *Muribaculaceae*, *Colidextribacter, Romboutsia*, *Lachnospiraceae-NK4A136-group, Acetatifactor, and Erysipelatoclostridium* (Figure 6B). In female mice, *Sphingomonas* and *Ralstonia* exhibited more pronounced associations (Figure 6D). Interestingly, a significant negative correlation between *Muribaculaceae* and NAFLD-related markers was observed in male mice, while no difference was found in female mice. The comparison of female and male groups demonstrated an inverse association among *Faecalibaculum*, *Coriobacteriaceae-UCG-002*, and *Bifidobacterium* as well as body weight, liver weight, and serum markers linked to NAFLD, indicating the potential benefits of these bacterial taxa.

### 2.7. Predicted Function of Intestinal Microbiota

**Figure 7:**
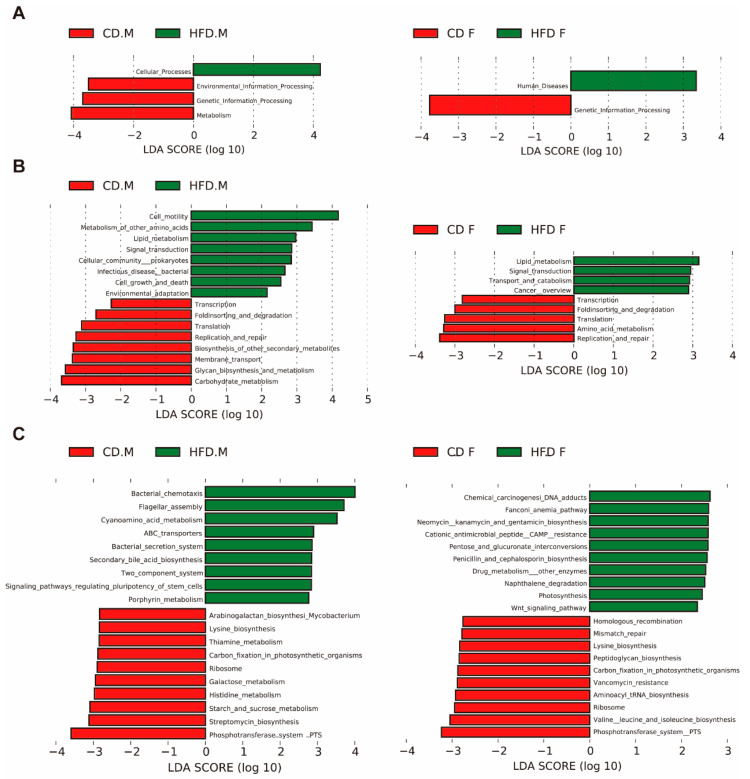
Functional differences of gut microbiota by HFD intervention. (**A**) KEGG pathway at the highest level of classification (level 1). (**B**) KEGG pathway at an intermediate level of classification (level 2). (**C**) KEGG pathway at an intermediate level of classification (level 3).

The function of gut microbiota was predicted using PICRUSt2 based on 16S rDNA sequences. To further investigate discrepancies in KEGG pathways among distinct groups, LEfSe was used to find differences in KEGG pathway abundances (Figure 7A–C). PICRUSt2 analysis showed that the functional genes predicted for gut microbiota after HFD intervention in the male group could be annotated to 4 primary pathways, 16 secondary pathways, and 19 tertiary pathways in the KEGG database. However, the female group could intervene in 2 primary pathways, 9 secondary pathways, and 20 tertiary pathways. In the HFD-induced male group, the most abundant pathways were related to cellular processes, metabolism, genetic information processing, and environmental information processing. The results of secondary prediction pathways showed that most of the predicted genes of cellular processes pathway were involved in cell motility, cell growth and death, and cellular community—prokaryotes. Further refining the cell motility pathway, an HFD upregulated the bacterial chemotaxis and flagellar assembly pathways. Most of the predicted genes in metabolic pathways are involved in metabolism of other amino acids, carbohydrate metabolism, glycan biosynthesis and metabolism, and lipid metabolism-related pathways. Further refinement revealed that HFD upregulated the cyanoamino acid metabolism, secondary bile acid biosynthesis, and fatty acid biosynthesis pathways and downregulated starch and sucrose metabolism, galactose metabolism, histidine metabolism, thiamine metabolism, and other metabolic pathways. In the female group, the identified bacterial genes may be associated with human diseases and genetic information processing, and the results of secondary prediction pathways showed that most of the predicted genes in the genetic information processing pathway were involved in replication and repair, translation, and transcription-related pathways, further refining our observation that an HFD inhibited the ribosome- and DNA replication- related pathways. Through analyzing the data of male and female groups, we found that HFD significantly inhibited the pathways of phosphotransferase system-PTS and ribosome.

### 2.8. HFD Affects the Integrity and Permeability of the Intestinal Epithelium

**Figure 8:**
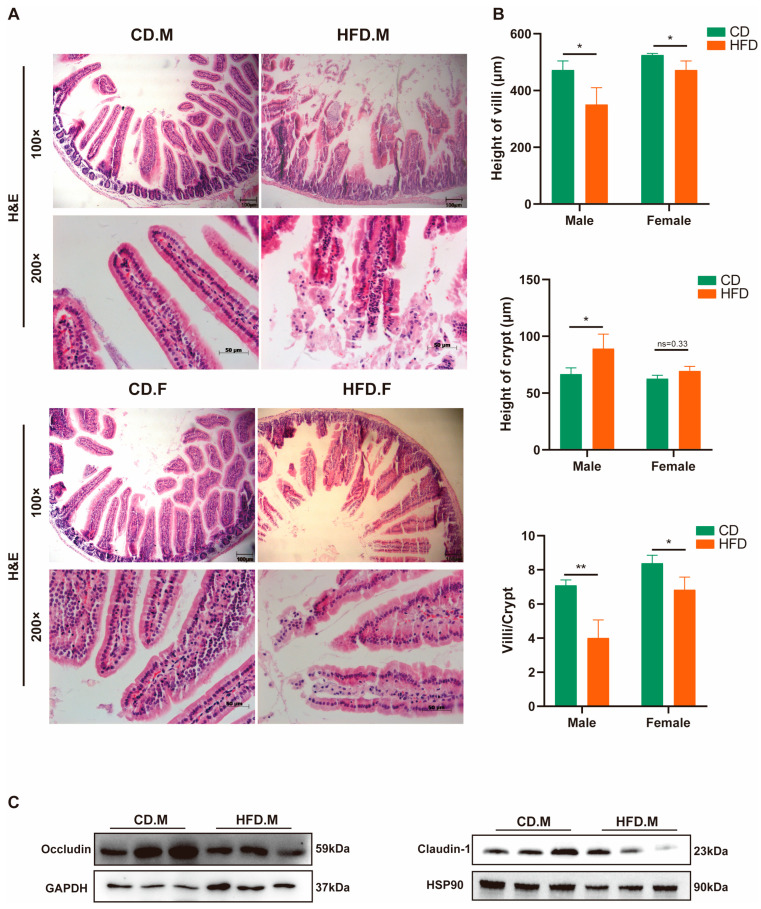
Effects of HFD on mice intestinal barrier. (**A**) H&E staining of mice intestinal tissue between the CD or HFD groups. Scale bar: 100 µm or 50 µm. (**B**) The villus height, crypt depth, and V/C ratio of the jejunum were compared between the CD or HFD groups. (**C**) The protein levels of Occludin and Claudin-1. * indicates *p*-value < 0.05, ** indicates *p*-value < 0.01, ns indicates *p*-value > 0.05 (*t*-test).

The presence of NAFLD has been associated with dysbiosis in the gut microbiota, inflammation, and impairment of the intestinal barrier. Histological analysis using H&E staining revealed that male and female mice fed with an HFD exhibited disruption of the epithelial layer, the presence of abscesses, increased edema, and cellular infiltration into the submucosal layer (Figure 8A). Furthermore, HFD mice showed a significant decrease in villus height (*p* < 0.05) and a significant increase in crypt depth (*p* < 0.05) in the cecum. Additionally, the ratio of villus height to crypt depth (V/C value) significantly decreased (*p* < 0.05) (Figure 8B), which is an important indicator of intestinal villus integrity and digestion and absorption capabilities. Meanwhile, HFD mice showed a significant decreased expression of the tight junction protein Occludin (Figure 8C). The results indicate that an HFD could affect the integrity of the intestinal epithelium, leading to changes in intestinal permeability and damage to the defense mechanisms of the intestinal mucosa.

## 3. Discussion

Non-alcoholic fatty liver disease (NAFLD) is characterized by perturbed lipid metabolism within the liver, and it prevails as the most prevalent liver ailment globally [19,20]. A mounting body of evidence suggests a link between gut dysbiosis and the onset of NAFLD [21]. Hence, we have successfully established murine models of NAFLD in both female and male subjects. Additionally, our model unveiled a gender-specific disparity in susceptibility to NAFLD, wherein female mice exhibited a certain degree of resistance towards an HFD, while male mice demonstrated significantly higher absolute weight, liver index, and blood biochemical indices, aligning with previous research findings [22,23,24]. Ovariectomized animal models demonstrate a causal relationship between estrogen deficiency and an increased susceptibility to the development of hepatic steatosis and steatohepatitis.

Scientific evidence suggests that aside from caloric restriction, there is no one dietary approach proven optimal to treat NAFLD [7]. However, the consumption of a Mediterranean diet enriched with omega-3 polyunsaturated fatty acids has demonstrated protective effects against dysbiosis induced by an HFD, as well as improvements in the composition of the gut microbiome [25,26]. HFD-induced obesity is known to be associated with a low ratio of *Bacteroidetes* to *Firmicutes* [27]; *Bacteroidota* dominates the gut ecosystem and is involved in absorbing and degrading host polysaccharides [28]. Meanwhile, our study found large numbers of *Proteobacteria* and *Desulfobacterota* colonizing the gut microbiota of HFD-group mice. Recent investigations indicate that an escalated prevalence of *Proteobacteria* could serve as a potential diagnostic marker for dysbiosis and increased disease risk [29,30], while others have found that an increased abundance of *Desulfovibrionaceae* can promote endotoxemia formation and participate in NAFLD pathogenesis [28,31,32]. The *Patescibacteria* in our model exhibit significant sexual dimorphism, as evidenced by the observed variation in relative abundance between genders on a high-protein diet [33]. Previous studies found that *Patescibacteria* is highly abundant in patients with hepatocellular carcinoma [34]. However, their potential involvement in the pathogenesis of NAFLD remains to be investigated.

Notably, the abundance of potential probiotics such as *Faecalibacterium*, *Dubosiella*, *Coriobacteriaceae-UCG-002*, and *Muribaculaceae* were significantly decreased, while the relative abundances of *Colidextribacter* and *Lachnospiraceae-NK4A136-group* were increased after an HFD. Certain strains of commensal bacteria that produce short-chain fatty acids (SCFA) are also recognized for their anti-inflammatory properties. According to previous studies, *Colidextribacter* and the *Lachnospiraceae-NK4A136-group* are considered to be bacteria that are beneficial for intestinal health [35,36]. The *Lachnospiraceae-NK4A136-group*, a taxon renowned for its butyrate production, plays a crucial role in maintaining the integrity of the murine intestinal barrier and exhibits remarkable anti-inflammatory properties [37,38]. The addition of fucoidan resulted in an increase in the relative abundance of *Colidextribacter*, which subsequently led to elevated levels of SCFA, particularly butyric acid [39]. The previous studies have demonstrated that the herbal formula AMC, consisting of a combination of eight herbs, enhances homeostasis model assessment of insulin resistance (HOMA-IR) and plasma triglyceride levels, which are closely associated with the enrichment of *Faecalibacterium* [40]. *Faecalibacterium* is considered a bioindicator of gastrointestinal disorders and has been [41] found to exhibit a positive correlation with the production of butyric acid/SCFA [40,42]. The inhibitory effect of *Dubosiella* on obesity in mice has been reported [43], while *Coriobacteriaceae-UCG-002* benefits the host through the production of essential amino acids and fermentation of dietary proteins [44]. Additionally, research suggests that *Coriobacteriaceae-UCG-002* may improve steatosis in NAFLD through its effect on sphingolipid metabolism pathways [45]. Previous studies have identified *Muribaculaceae* as bacteria capable of degrading carbohydrates. However, it has been observed that an HFD decreases the abundance of this bacteria [46,47]. It suggests that *Muribaculaceae* plays a vital role in modulating energy metabolism in mice. The probiotic *Bifidobacterium* exhibits promising potential in mitigating diet-induced NAFLD [48].

Furthermore, our study indicates that the relative abundance of harmful bacteria, such as *Erysipelatoclostridium, Romboutsia,* and *Acetatifactor*, was significantly increased after an HFD, which aggravated the risk of host inflammation. Preliminary investigations suggest that *Erysipelatoclostridium ramosum* may play a key role in fluoride-induced obesity in mice [49,50]. *Ralstonia* have been shown to cause infections, even more serious ones, such as osteomyelitis and meningitis [45]. The pathogenic microbe *Acetatifactor* has been specifically identified as a contributor to dextran sulfate sodium (DSS)-induced colitis in mice [51]. Recent studies have successfully isolated *Acetatifactor muris* from obese mice on a high-calorie diet [52]. These altered bacteria may contribute to NAFLD progression; however, their specific mechanisms in NAFLD remain unclear.

Following intervention with an HFD, gut microbiota function was predicted to be significantly enriched in pathways such as bacterial chemotaxis, flagellar metabolism, cyanogenic amino acids, and ABC transporters. We hypothesize that an HFD may enhance pathogen adhesion through increasing bacterial chemotaxis activity and the functional activity of gut microbiota pathways related to flagellar assembly [53,54]. Additionally, the upregulation of functional activity in ABC transporters may increase the risk of pathogen colonization and inflammation [55]. Conversely, pathways such as the phosphotransferase system (PTS) and ribosome and starch/sucrose metabolism were found to be reduced. We hypothesize that PTS is likely to play an important role in glucose uptake and its regulation during NAFLD [56].

The findings of this study illustrate that the consumption of an HFD compromises the integrity of the intestinal barrier and disrupts glucose homeostasis [57], while the dysregulation of intestinal epithelial barrier function and microbial flora might contribute to the development of non-alcoholic steatohepatitis (NASH) [58]. Previous studies indicate that estrogens exert beneficial effects only with an HFD, but not normal chow [59,60]. Interestingly, we observed a reduction in intestinal damage among female mice on the HFD.

**Figure 9:**
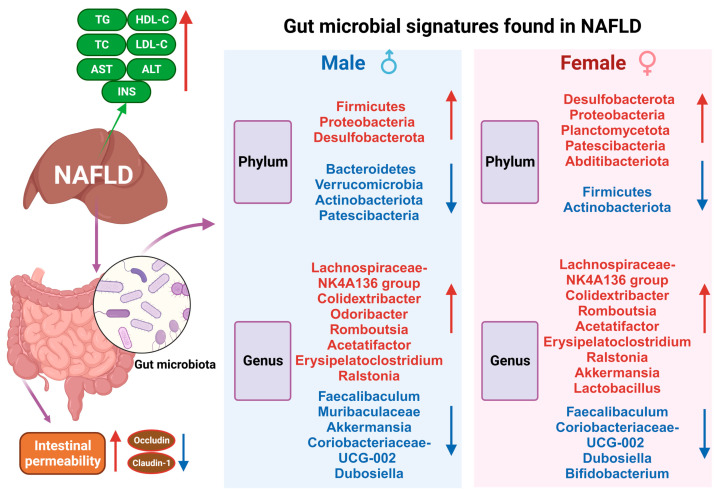
Gut microbial signatures found in NAFLD. The red arrows depict the upregulated gut microbes, while the blue arrows represent the down-regulated gut microbes.

In conclusion, our results reveal the composition and predictive function of gut microbiota in NAFLD models and altered gut microbiota were associated with serum parameters associated with NAFLD (Figure 9). Our data provide a new reference for the study of gut bacteria and fecal bacterial transplantation. There is no doubt that we have made significant progress in the analysis of the gut microbiota. However, the complex mechanisms underlying the interactions between the gut microbiome and the host need to be further explored.

## 4. Materials and Methods

### 4.1. Animal Feeding

Six-week-old specific pathogen-free C57BL/6 mice (male, 18 to 20 g) (female, 15 to 18 g) were housed in an appropriate temperature-controlled room (22 ± 2 °C) with a 12 h light–dark cycle (ON at 8:00 a.m., OFF at 8:00 p.m.). After one week of feeding, mice were randomly selected and divided into four groups: male CD group, female CD group (*n* = 10, chow diet, CD), male HFD group, and female HFD group (*n* = 10, high-fat diet, HFD) (Figure 1A). The experimental mice were fed a refined high-fat diet (XTHF60), which contained 20% carbohydrate, 20% protein, and 60% fat with a total energy of 5.24 kcal/g, and the CD group was fed a refined chow diet (XTCON50J), which contained 70% carbohydrate, 20% protein, and 10% fat with a total energy of 3.85 kcal/g. All experimental mice had free access to food and water. The physical activity, food and water consumption, and feces of the experimental mice were observed daily. HFD and CD were purchased from Jiangsu Xietong Pharmaceutical Bio-engineering Co., Ltd. Nanjing, China.

The animal study protocol was approved by the Institutional Animal Care and Use Committee (IACUC) of the Animal Experimental Ethics Committee of Yangzhou University (Permit Number: SYXK (Su) 2022-0044). All experimental methods and processes were performed according to the relevant regulations.

### 4.2. Sample Collection

The body weights of mice were recorded every two weeks, and samples were collected 16 weeks later. At the end of the prescribed feeding period, all mice were fasted overnight and anesthetized with an intraperitoneal injection of 1% pentobarbital sodium (50 mg/kg body weight). Blood samples were collected from the cardiac artery. The liver samples were dissected and weighed immediately. The liver index was calculated using the following formula: liver wet weight/total body weight × 100%. Mouse hepatic and small intestinal samples were fixed in 4% neutral buffered formalin and embedded in paraffin. Paraffin-embedded samples were sectioned and then subjected to hematoxylin and eosin (H&E) staining for assessment of liver and intestine histopathology. Oil Red O staining was performed on the frozen sections to evaluate the hepatic steatosis. The contents of the cecum were placed in liquid nitrogen and tested for the microbiome.

### 4.3. Biochemical Analysis of Serum

Blood specimens were allowed to clot naturally for 2 h at room temperature and then centrifuged at 1000× *g* for 15 min. Supernatants were collected for serum-related parameters. Serum alanine aminotransferase (ALT), aspartate aminotransferase (AST), total cholesterol (TC), triacylglycerol (TG), high-density lipoprotein cholesterol (HDL-C), and low-density lipoprotein cholesterol (LDL-C) were analyzed using an automatic biochemical analyzer (BS-420 Mindray, Shenzhen, China). Levels of insulin (INS) were determined using an enzyme-linked immunosorbent assay (ELISA) technical kit (HY-D0001, Huaying, China) according to the manufacturer’s protocol.

### 4.4. Gut Microbiota Sequencing

Total DNA was extracted from intestinal contents (cecum and colon) using a Fast DNA Stool Kit (Cat# DP328, TIANGEN Biotech Co. Ltd., Bejing, China) and used for PCR amplification of the v3–v4 regions of the 16S rRNA gene with barcoded primers 341F and 806R. The PCR amplicons were purified and sequenced on the Illumina NovaSeq platform (Illumina, San Diego, CA, USA) by Novogene (Novogene, Beijing, China). The composition and function of the gut microbiome were assessed using Quantitative Insights Into Microbial Ecology2 [61] and Tax4Fun2 [62].

### 4.5. Western Blot Analysis

This section was performed in accordance with the former studies. Briefly, proteins from jejunum tissues were extracted via sonication in RIPA lysis buffer with phenylmethanesulfonylfluoride. After centrifugation at 12,000× *g* rpm for 20 min at 4 °C, the supernatant of the lysate was collected, and the protein concentration was detected using the BCA method. The proteins of samples were fractionated through 10% SDS-PAGE and transferred onto NC membranes. After being blocked with 5% non-fat milk for 2 h at room temperature, TBST washing film was used three times (10 min each), and the membranes were incubated with primary antibodies overnight at 4 °C. After washing with TBST three times (10 min each), the blots were incubated with goat anti-rabbit IgG antibody (Abcam, Cambridge, UK, ab205718) or anti-mouse IgG antibody (HuaBio, Woburn, MA, USA HA1006) for 2 h at room temperature. Finally, the signal was visualized using ECL reagent. Western blot quantification was analyzed using ImageJ (1.8.0) software and normalized to HSP90. The antibodies against HSP90 (60318-1-lg), Occludin (27260-1-AP), and Claudin 1 (13050-1-AP) were procured from Proteintech Ltd. (Wuhan, China).

### 4.6. Statistical Analysis

Data are presented as mean ± SD. The statistical significance of body weight, liver weight, liver index, serum indexes (TC, TG, HDL, LDL, AST, and ALT), and the relative abundance of gut microbiota were determined with Student’s *t*-test or the Wilcoxon rank sum test. A *p*-value < 0.05 was considered statistically significant. Statistical analysis was performed with SPSS software (Version 22, SPSS Inc., Chicago, IL, USA). Microbial gene function was predicted using PICRUSt2 (Version 2.1.4) software. Predicted genes and their functions were then annotated using the Kyoto Encyclopedia of Genes and Genomes (KEGG) database.

## Data Availability

The data presented in this study can be obtained upon request from the corresponding author. The data are not publicly accessible due to their inclusion of other topics.
